# The end-expiratory occlusion test for detecting preload responsiveness: a systematic review and meta-analysis

**DOI:** 10.1186/s13613-020-00682-8

**Published:** 2020-05-24

**Authors:** Francesco Gavelli, Rui Shi, Jean-Louis Teboul, Danila Azzolina, Xavier Monnet

**Affiliations:** 1 Service de Médecine Intensive-Réanimation, Hôpital de Bicêtre, Hôpitaux Universitaires Paris-Saclay, 78, rue du Général Leclerc, 94270 Le Kremlin-Bicêtre, France; 2 Université Paris-Saclay, Faculté de Médecine Paris-Saclay, Inserm UMR S_999, 94270 Le Kremlin-Bicêtre, France; 3 Emergency Medicine Unit, Department of Translational Medicine, Università degli Studi del Piemonte Orientale, 28100 Novara, Italy; 4 Department of Translational Medicine, Università degli Studi del Piemonte Orientale, 28100 Novara, Italy

**Keywords:** Heart–lung interactions, Haemodynamic monitoring, Fluid responsiveness, Cardiac preload, Fluid challenge

## Abstract

**Background:**

We performed a systematic review and meta-analysis of studies assessing the end-expiratory occlusion test (EEXPO test)-induced changes in cardiac output (CO) measured by any haemodynamic monitoring device, as indicators of preload responsiveness.

**Methods:**

MEDLINE, EMBASE and Cochrane Database were screened for original articles. Bivariate random-effects meta-analysis determined the Area under the Summary Receiver Operating Characteristic (AUSROC) curve of EEXPO test-induced changes in CO to detect preload responsiveness, as well as pooled sensitivity and specificity and the best diagnostic threshold.

**Results:**

Thirteen studies (530 patients) were included. Nine studies were performed in the intensive care unit and four in the operating room. The pooled sensitivity and the pooled specificity for the EEXPO test-induced changes in CO were 0.85 [0.77–0.91] and 0.88 [0.83–0.91], respectively. The AUSROC curve was 0.91 [0.86–0.94] with the best threshold of CO increase at 5.1 ± 0.2%. The accuracy of the test was not different when changes in CO were monitored through pulse contour analysis compared to other methods (AUSROC: 0.93 [0.91–0.95] vs. 0.87 [0.82–0.96], respectively, *p* = 0.62). Also, it was not different in studies in which the tidal volume was ≤ 7 mL/kg compared to the remaining ones (AUSROC: 0.96 [0.92–0.97] vs. 0.89 [0.82–0.95] respectively, *p* = 0.44). Subgroup analyses identified one possible source of heterogeneity.

**Conclusions:**

EEXPO test-induced changes in CO reliably detect preload responsiveness. The diagnostic performance is not influenced by the method used to track the EEXPO test-induced changes in CO.

*Trial registration* The study protocol was prospectively registered on PROSPERO: CRD42019138265.

## Background

Over the last 20 years, many dynamic tests were developed and validated to predict whether a fluid bolus will increase cardiac output (CO) significantly [1]. They all consist in observing the effects on CO of variations in cardiac preload occurring under different circumstances. The variations of arterial pulse pressure and stroke volume induced by mechanical ventilation are very reliable indices of preload responsiveness [2, 3], but they are strongly limited by the restricted conditions in which they can be used. Administering small amounts of fluid may predict the response to larger ones [4], but such “mini fluid challenges” require a very precise measurement of CO and, if repeated, may contribute to fluid overload. Passive leg raising reversibly mimics fluid infusion and detects preload responsiveness very reliably [5], but intra-abdominal hypertension is responsible for some false-negatives [6] and it is not very convenient to perform [7].

In this context, the transient interruption of mechanical ventilation at end-expiration was recommended 10 years ago for testing preload responsiveness through heart–lung interactions [8]. By interrupting the impediment to venous return induced by each mechanical insufflation, the expiratory hold allows the cardiac preload to augment, which, in case of preload responsiveness, leads to a significant increase of CO [9].

Some studies testing the diagnostic accuracy of the end-expiratory occlusion (EEXPO) test have been published after that first one, with different methods of CO measurement, durations of expiratory hold and clinical settings. A meta-analysis [10] has been performed with eight of these studies [8, 11–17]. However, it failed to include two studies [18, 19] which had already been published about the reliability of the EEXPO test. Moreover, no subgroup analysis was performed to look for factors of heterogeneity, whilst some of them might be significant. This might be the case, for instance, for the duration of the EEXPO or the technique used to monitor CO [10]. Finally, some additional studies [20–22] were published afterwards, and additional patients may allow one to perform the subgroup analysis that had not been performed by Messina et al. [10]. Then, we conducted a new systematic review of all the studies testing the diagnostic accuracy of the EEXPO test. In particular, taking advantage of the large number of patients pooled, we aimed at looking for factors influencing the reliability of the EEXPO test.

## Methods

### Clinical research question

The clinical research question was: What is the sensitivity and specificity of the EEXPO test to detect preload responsiveness when its effects are assessed on cardiac output?

### PICO statement

The PICO statement was the following:

- P—patient, problem or population: surgical or critically ill patients under mechanical ventilation in whom the effect of volume expansion on CO needs to be predicted.
- I—intervention: EEXPO test performed by holding the patient’s breath at the end of expiration during invasive mechanical ventilation and by measuring the induced changes in CO, measured by any available monitoring device.
- C—comparison, control or comparator: preload responsiveness defined as either a 10 to 15% increase in CO during volume expansion (250–500 mL of fluid in ≤ 30 min) or 10% during passive leg raising (PLR), measured by any available monitoring devices.
- O—outcomes: ability of the EEXPO test to detect preload responsiveness, defined in each study according to the pre-specified threshold of CO increase after either volume expansion or PLR.

### Identification of records

Our aim was to identify all studies evaluating the ability of the EEXPO test to predict a significant increase in CO or surrogate (velocity time integral of the left ventricular outflow tract with echocardiography, blood velocity of the descending aorta with oesophageal Doppler) compared to the one induced by a subsequent volume expansion or by a PLR test. We included into our analysis only the studies that provided sensitivity, specificity and the area under the receiver operating characteristic curve (AUROC) of the EEXPO test with the corresponding diagnostic threshold. Moreover, only studies on adults, that were published in full text or accepted for publication in indexed journals, were included in our analysis. No language restriction was applied.

We searched the US National Library of Medicine’s MEDLINE database, the EMBASE database and the Cochrane Database of Systematic Reviews for relevant studies published from 1960 to 1st October 2019. We used the following medical subject headings and keywords: “end expiratory occlusion”, “end expiratory”, “volume expansion”, “fluid challenge”, “fluid administration”, “fluid responsiveness”, “preload responsiveness”. The complete searching strategy is reported in Additional file 1: Figure S1. We also looked for relevant articles cited in reviews, articles and editorials. The search was performed by two independent investigators (FG and RS) until no new record could be found. Conflicts regarding inclusion or exclusion of studies were resolved by consensus with a third investigator (XM). The meta-analysis was performed according to the PRISMA statement (http://www.prisma-statement.org). The study protocol was prospectively registered in PROSPERO (CRD42019138265—Submission 7th June 2019, approval 29th August 2019).

### Data extraction

Using a standardised form, two investigators (FG and SR) independently extracted several data from the selected studies, including demographic characteristics of the investigated population, ventilatory variables, the duration of the EEXPO test, the method used to assess its haemodynamic effects on CO or its surrogate, the amount and type of fluid infused and the duration of the infusion of volume expansion, when performed, as well as the criteria used to define preload responsiveness. Moreover, the number of true-positives, true-negatives, false-positives and false-negatives as well as sensitivity and specificity, the AUROC and the best EEXPO-induced increase in CO or surrogates able to detect preload responsiveness were collected.

### Assessment of risk of bias in included studies

Two authors (FG and RS) independently assessed the overall quality of evidence at the outcome level according to the GRADE system [23]. Moreover, they assessed the risk of bias of the included studies by following the criteria specified in the QUADAS-2 scale [24]. For each criterion, the risk of bias was judged as high, low or unclear. Disagreements between the reviewers were resolved by consensus with a third investigator (XM). Then, as described elsewhere [5], points were given to each issue of the QUADAS-2 evaluation (three points for “high”, two points for “unclear” and one point for “low”) and their sum was calculated. “Overall higher” and “overall lower” risk of bias was defined with reference to the median of the risk bias of all studies [5].

### Statistical analysis

#### Study description

Study-specific sensitivity and specificity values have been computed considering a 0.5 continuity correction as indicated in the literature (Additional file 1: Figure S2). The 95% confidence intervals have also been calculated using the Wilson [25] method. A graphical representation of the data has been provided. Paired forest plots on sensitivity and specificity and confidence ellipses (95%) plots have also been reported. The correlation of sensitivities and false-positive rates has been reported to investigate a possible threshold effect.

For the principal analysis, if more than one technique was used to assess the haemodynamic effects of the EEXPO test, we chose the one considered to be the most reliable: when both oesophageal Doppler and end-tidal carbon dioxide were used, we only considered oesophageal Doppler and when both echocardiography or oesophageal Doppler and calibrated pulse contour analyses were used, we considered only pulse contour analysis. Finally, for that analysis, in studies in which the EEXPO test was performed at different positive end-expiratory pressure (PEEP) or tidal volume levels, we selected the ones that provided the highest AUROC.

#### Bivariate random-effect model

The bivariate random-effects model by Reitsma [26] was computed to estimate the area under the summary receiver operating characteristic (AUSROC) curve accounting for correlation between sensitivity and specificity. The model was estimated through a restricted maximum likelihood approach. In the bivariate model, the logit sensitivity and the logit specificity are assumed to be bivariate normal random variables across the studies considering also a variance and covariance matrix for the random-effect component. A bivariate version of *I*^2^ statistics was computed to investigate the presence of heterogeneity on sensitivity and specificity outcome, as indicated in the literature [27]. A value of *I*^2^ ≥ 75% was considered as indicating a high heterogeneity [28].

#### Investigation of heterogeneity sources

The potential sources of heterogeneity were investigated considering a Reitsma bivariate random-effect meta-regression model. Separate meta-regression models were calculated, considering as covariates:

- Tidal volume: ≤ 7 vs. > 7 mL/kg.
- Pulse contour analysis vs. other haemodynamic monitoring methods.
- EEXPO duration: ≤ 15 vs. > 15 s.
- PEEP level: ≤ 7 vs. > 7 cmH_2_O.
- Setting of the study: intensive care unit (ICU) vs. operating room (OR).
- Risk of bias: “overall lower” vs. “overall higher”, as described above.

The covariate effects on the sensitivity and false-positive rate were reported together with *p* values and 95% confidence intervals. The likelihood ratio test was carried out comparing a null model with a model with a covariate. A significant likelihood ratio test indicates that the covariate is a potential source of heterogeneity across studies. Publication bias was investigated using the Deeks’s test [29]. The statistical significance was set at a *p* value < 0.05. The analyses were performed using R 3.3.5 [30] with mada package [31].

## Results

### Characteristics of the included studies

**Fig. 1:**
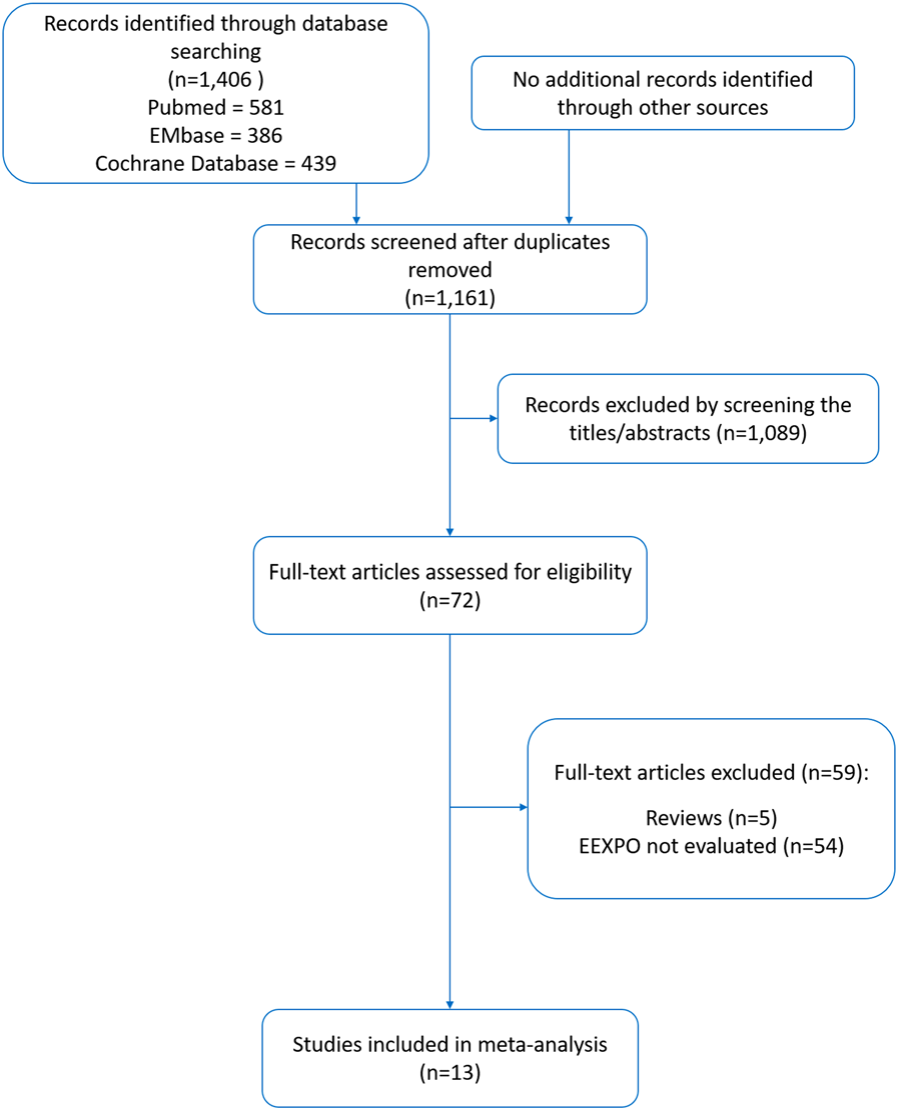
Flowchart of study selection

We identified 13 studies (530 patients) [8, 11–22] that reported the ability of the EEXPO test to assess preload responsiveness. The flowchart in Fig. 1 illustrates the study selection and the main characteristics of the included studies reported in Table 1. Nine studies [8, 11, 14–20] were performed in the ICU and four in the OR [12, 13, 21, 22]. In one study in the ICU [15], the EEXPO test was performed during prone positioning. All patients were mechanically ventilated with a tidal volume ranging between 5.8 mL/kg [20] and 8.2 mL/kg [12], with a median value of 6.95 mL/kg. In two studies [14, 21], the diagnostic ability of the EEXPO test was assessed under a tidal volume at 6 mL/kg and repeated at a tidal volume at 8 mL/kg. The PEEP level was set between 4 cmH_2_O [12] and 14 cmH_2_O [19], with a median value of 7 cmH_2_O. The results of the QUADAS-2 evaluation are reported in Additional file 1: Figure S3. Following the GRADE system, the overall quality of evidence for the included studies was assessed as very low (Additional file 1: Figure S4).

**Table 1:** Studies characteristics

	Year	No. of patients	Setting	PEEP (cmH_2_O)	Tidal volume (mL/kg)	Respiratory system compliance (mL/cmH_2_O)	HD monitoring #1	HD monitoring #2
Monnet et al. [8]	2009	34	ICU	8 ± 3	6.8 ± 1.1	*NA*	Calibrated PC	PP
Monnet et al. [18]	2012	39	ICU	7 ± 3	7.9 ± 2.5	36 ± 16	Calibrated PC	*NA*
Monnet et al. [11]	2012	54	ICU	7 ± 3	7.9 ± 1.1	33 ± 6	Calibrated PC	*NA*
Silva et al. [19]	2013	34	ICU	5 ± 0 vs. 14 ± 0	6.8 ± 0.2	27 ± 3 vs. 31 ± 1	Calibrated PC	*NA*
Guinot et al. [12]	2014	42	OR	4 ± 2	8.2 ± 0.8	*NA*	ODM	EtCO_2_
Biais et al. [13]	2017	41	OR	5 ± 0	6.9 ± 0.6	40 ± 10	Uncalibrated PC	*NA*
Myatra et al. [14]	2017	30	ICU	9 ± 3	6.0 ± 0.1 vs. 8.0 ± 0.0	25 ± 4 vs. 32 ± 4	Calibrated PC	*NA*
Yonis et al. [15]	2017	33	ICU	8 ± 1	6.0 ± 0.1	30 ± 5	Calibrated PC	*NA*
Jozwiak et al. [16]	2017	30	ICU	10 ± 4	6.2 ± 0.2	35 ± 3	TTE	Calibrated PC
Georges et al. [17]	2018	50	ICU	6 ± 2	6.9 ± 0.7	50 ± 17	TTE	*NA*
Dépret et al. [20]	2019	28	ICU	12 ± 3	5.8 ± 0.6	39 ± 10	ODM	Calibrated PC
Messina et al. [21]	2019	40	OR	5 ± 0	6.0 ± 0.0 vs. 8.0 ± 0.0	65 ± 4 vs. 83 ± 4	Uncalibrated PC	*NA*
Xu et al. [22]	2019	75	OR	5 ± 0	8 ± 0.1	*NA*	TOE	*NA*

*EtCO*_*2*_ end-tidal carbon dioxide, *HD* haemodynamic, *ICU* intensive care unit, *NA* not available, *ODM* oesophageal Doppler monitoring, *OR* operating room, *PC* pulse contour, *PEEP* positive end-expiratory pressure, *TOE* trans-oesophageal echocardiography, *TTE* trans-thoracic echocardiography

### Haemodynamic monitoring

Four studies provided more than one method for CO measurement [8, 12, 16, 20]. In eight of the included studies [8, 11, 14–16, 18–20] CO was evaluated through the calibrated pulse contour analysis and in two through the uncalibrated one [13, 21]. Three studies [16, 17, 22] evaluated the effects of EEXPO test on CO with echocardiography: two with transthoracic [16, 17] and one with transoesophageal echocardiography [22]. Oesophageal Doppler was used in two studies [12, 20], end-tidal carbon dioxide monitoring [12] and pulse pressure [8] in one study each.

### Fluid responsiveness

Preload responsiveness was defined according to CO changes induced by fluid administration in 12 studies [8, 11–18, 20–22]. In these studies, preload responsiveness was defined by a fluid-induced increase in CO ≥ 15% [8, 11, 12, 14–18, 20, 22] or 10% [13, 21]. Preload responsiveness was defined according to CO changes induced by PLR in one study, with a threshold of CO increase of 10% [19].

Fluid infusion was performed with normal saline in 11 studies [8, 11, 13–20, 22], with Ringer solution in the other two studies [12, 21], with infused volumes of 500 mL in most of the cases [8, 11, 12, 15–20]. However, in two studies [13, 21] the volume of the fluid bolus was 250 mL and in two others it was tailored according to patient’s body weight [14, 22] (Table 2).

**Table 2:** Modalities of the end-expiratory occlusion test and of fluid

	Year	No. of patients	Responders	Non-responders	FC duration (min)	FC volume (mL)	Reference defining preload responsiveness	CO increase defining responsiveness (%)	EEXPO duration (s)
Monnet et al. [8]	2009	34	23	11	10	500	Saline infusion	15	15
Monnet et al. [18]	2012	39	17	22	30	500	Saline infusion	15	15
Monnet et al. [11]	2012	54	30	24	20	500	Saline infusion	15	15
Silva et al. [19]	2013	34	13	21	–^a^	–^a^	PLR	10	15
Guinot et al. [12]	2014	42	28	14	10	500	Ringer/ringer lactate infusion	15	15
Biais et al. [13]	2017	41	20	21	10	250	Saline infusion	10	30
Myatra et al. [14]	2017	30	16	14	10	7 mL/kg	Saline infusion	15	15
Yonis et al. [15]	2017	33	15	18	15	500	Saline infusion	15	15
Jozwiak et al. [16]	2017	30	15	15	10	500	Saline infusion	15	15
Georges et al. [17]	2018	50	28	22	15	500	Saline infusion	15	12
Dépret et al. [20]	2019	28	14	14	10	500	Saline infusion	15	15
Messina et al. [21]	2019	40	21	19	10	250	Ringer lactate infusion	10	30
Xu et al. [22]	2019	75	36	39	10	6 mL/kg	Saline infusion	15	20

*CO* cardiac output, *EEXPO* end-expiratory occlusion, *FC* fluid challenge, *PLR* passive leg raising

^a^In this study, a fluid challenge was performed in some patients, but preload responsiveness was defined according to the result of the PLR test, which was performed in all the patients

### Prediction of fluid responsiveness by the EEXPO test-induced changes in CO

The duration of the expiratory hold was reported in all the included studies and it ranged between 12 s [17] and 30 s [13, 21]. All the studies reported the AUROC curve for the EEXPO test to detect preload responsiveness [8, 11–22], as well as sensitivity, specificity and the best diagnostic threshold (Table 3).

**Table 3:** Diagnostic accuracy of the end-expiratory occlusion test in the including studies

	No. of patients	AUROC	95% CI	Threshold (%)^a^	Sensitivity (%)	Specificity (%)	PPV (%)	NPV (%)
Monnet et al. [8]	34	0.97	0.85–1.00	5	91	100	100	84
Monnet et al. [18]	39	0.97	0.91–1.00	5	100	91	90	100
Monnet et al. [11]	54	0.95	NA	5	93	92	94	91
Silva et al. [19]	34	0.96	0.82–0.99	6	100	90	86	100
Guinot et al. [12]	42	0.78	0.63–0.89	2.3	82	71	85	66
Biais et al. [13]	41	0.91	0.81–1.00	5	100	81	83	100
Myatra et al. [14]	30	0.95	0.88–1.00	4.1	88	93	93	87
Yonis et al. [15]	33	0.65	0.46–0.84	10	33	100	100	64
Jozwiak et al. [16]	30	0.98	0.85–1.00	4	93	100	100	93
Georges et al. [17]	50	0.96	NA	9	89	95	96	87
Dépret et al. [20]	28	0.95	0.79–0.99	3	86	93	92	87
Messina et al. [21]	40	0.93	0.84–1.00	3.6	89	86	87	88
Xu et al. [22]	75	0.9	0.83–0.97	5	81	93	91	84

*AUROC* area under the receiver operating characteristic, *CI* confidence interval, *NA* not available, *NPV* negative predictive value, *PPV* positive predictive value

^a^Threshold of increase in cardiac output induced by the test reported as providing the best compromise between sensitivity and specificity

**Fig. 2:**
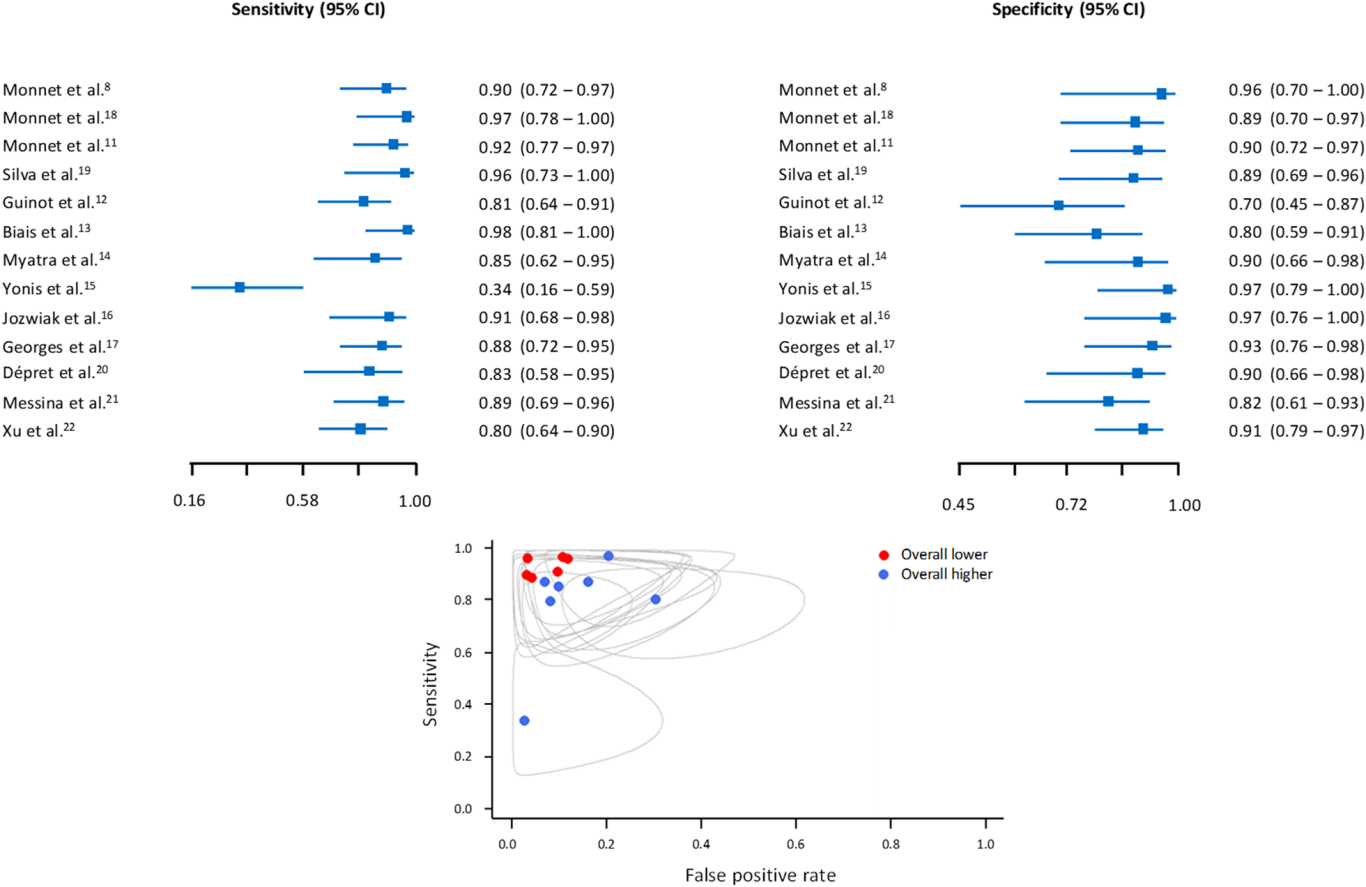
Paired sensitivity and specificity forest plots (top) and confidence ellipses plot (bottom) according to “Overall lower” and “Overall higher” QUADAS-2 risk of bias. The Spearman correlation of sensitivities and false-positive rates is 0.27 [− 0.32 to 0.72]

**Fig. 3:**
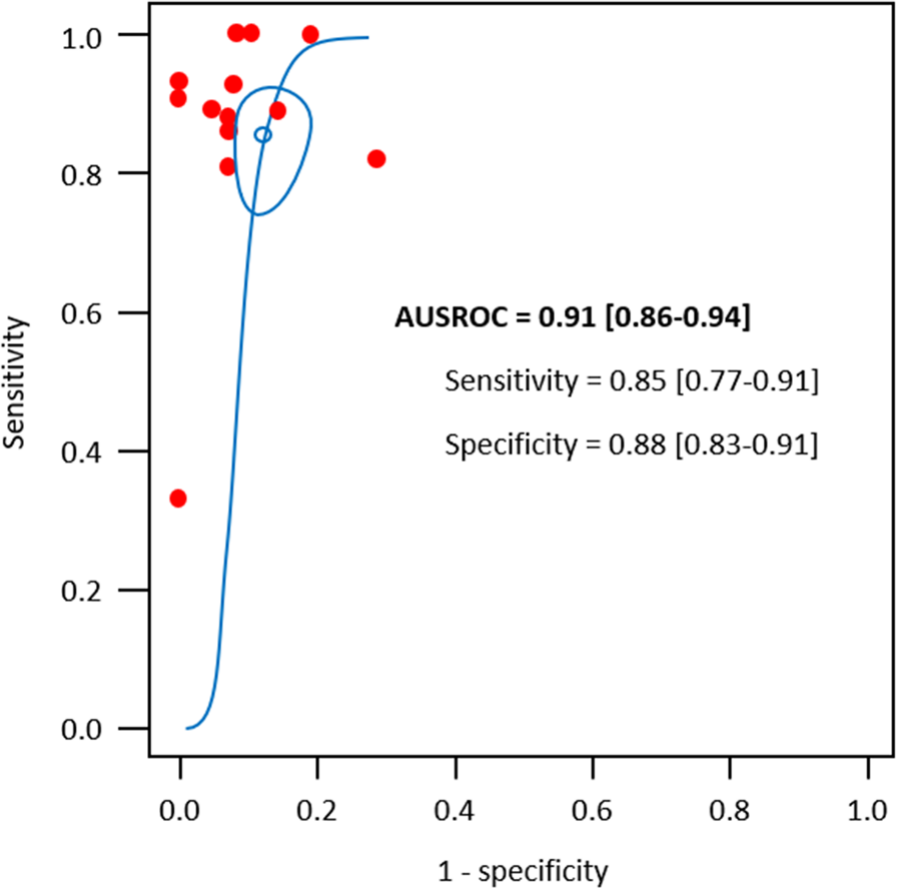
AUSROC curve for the Reitsma et al. [26] bivariate model. Pair of pooled accuracies together with a 95% confidence region is represented. AUSROC: area under the summary receiver operating characteristic

For the EEXPO test-induced changes in CO, the pooled sensitivity and specificity were 0.85 [0.77–0.91] (*I*^2^ = 62.6%) and 0.88 [0.83–0.91] (*I*^2^ = 6.0%), respectively, whilst the AUSROC curve was 0.91 [0.86–0.94] (Figs. 2 and 3). The corresponding best diagnostic threshold was 5.1 ± 0.2%. The Spearman correlation of sensitivities and false-positive rates was 0.27 [0.32–0.72].

### High vs. low tidal volume

For the nine studies with a tidal volume ≤ 7 mL/kg [8, 13–17, 19–21], the AUSROC curve was 0.96 [0.92–0.97] (sensitivity and specificity 0.89 [0.70–0.96] and 0.92 [0.83–0.96], respectively), whilst in the six studies with a tidal volume > 7 mL/kg [11, 12, 14, 18, 21, 22], it was 0.89 [0.82–0.95] (sensitivity and specificity 0.85 [0.78–0.90] and 0.87 [0.78–0.92], respectively). No significant difference was observed between pooled AUSROCs (*p* = 0.44) (Additional file 1: Figure S5.1).

### Pulse contour analysis vs. other haemodynamic monitoring techniques

Amongst the ten studies in which CO was measured through pulse contour analysis [8, 11, 13–16, 18–21], the AUSROC curve was 0.93 [0.91-0.95] (sensitivity and specificity 0.87 [0.75–0.94] and 0.89 [0.83–0.93], respectively), whilst amongst those that measured it through other methods [8, 12, 16, 17, 20, 22], it was 0.87 [0.82–0.96] (sensitivity and specificity 0.84 [0.78–0.89] and 0.88 [0.72–0.95], respectively). The comparison between the two AUSROCs did not show a significant difference (*p* = 0.62). (Additional file 1: Figure S5.2).

### EEXPO test duration

Amongst the ten studies in which the duration of the EEXPO test was ≤ 15 s [8, 11, 12, 14–20], the AUSROC curve was 0.93 [0.90–0.96] (sensitivity and specificity 0.86 [0.75–0.93] and 0.89 [0.83–0.94], respectively), whilst amongst those in which the EEXPO test duration was > 15 s [13, 21, 22], it was 0.93 [0.88–0.95] (sensitivity and specificity 0.87 [0.72–0.95] and 0.86 [0.74–0.93], respectively). There was no statistically significant difference between the two AUSROCs (*p* = 0.20) (Additional file 1: Figure S5.3).

### PEEP level

Amongst the eight studies [11–13, 17–19, 21, 22] in which the level of PEEP was ≤ 7 cmH_2_O, the AUSROC curve was 0.89 [0.83–0.95] (sensitivity and specificity 0.86 [0.80–0.91] and 0.86 [0.79–0.91], respectively), whilst amongst those in which the PEEP level was > 7 cmH_2_O [8, 14–16, 19, 20], it was 0.95 [0.92–0.97] (sensitivity and specificity 0.85 [0.62–0.95] and 0.93 [0.85–0.97], respectively). There was no statistically significant difference between the two AUSROCs (*p* = 0.386) (Additional file 1: Figure S5.4).

### Setting

Amongst the nine studies performed in the ICU [8, 11, 14–20], the AUSROC curve was 0.95 [0.93-0.96] (sensitivity and specificity 0.88 [0.74–0.95] and 0.92 [0.87–0.96], respectively), whilst amongst those performed in the OR [12, 13, 21, 22], it was 0.86 [0.82–0.93] (sensitivity and specificity 0.83 [0.74–0.90], and 0.83 [0.71–0.90], respectively). There was no statistically significant difference between the two AUSROCs (*p* = 0.66) (Additional file 1: Figure S5.5).

### Risk of bias

When we divided the studies according to the global risk of bias, no significant difference was observed in AUSROCs between studies with overall lower [8, 11, 16, 18–20] (0.96 [0.92–0.97]; sensitivity and specificity 0.92 [0.85–0.96] and 0.91 [0.84–0.95], respectively) and overall higher [12–15, 17, 21, 22] risk of bias (0.91 [0.83–0.95]; sensitivity and specificity 0.81 [0.65–0.91] and 0.87 [0.78–0.93], respectively) (*p* = 0.45) (Additional file 1: Figure S5.6).

### Sources of heterogeneity and publication bias

In the Reitsma bivariate random-effect meta-regression models, only the overall risk of bias emerged as a potential source of heterogeneity (*p* = 0.049) (Additional file 1: Figure S5.6). On the contrary, none of the other covariates was identified as a source of heterogeneity. According to the results of the Deeks’s test, we did not detect publication bias in the studies that evaluated the diagnostic performance of the EEXPO test (*p* = 0.864) (Additional file 1: Figure S6).

## Discussion

This meta-analysis of 13 studies performed in 530 patients shows that the changes in CO induced by the EEXPO test reliably detect preload responsiveness with excellent sensitivity and specificity (0.85 [0.77–0.91] and 0.88 [0.83–0.91], respectively). The AUSROC curve was 0.91 [0.86–0.94] and the best diagnostic threshold for the EEXPO-induced increase in CO was 5.1 ± 0.2%. No difference was observed for the diagnostic ability of the EEXPO test when different conditions, settings and methods for CO monitoring were compared.

The EEXPO test is based on heart–lung interactions. During positive pressure ventilation, insufflation increases the intrathoracic pressure and right atrial pressure, impeding venous return [2]. It interrupts the increase in cardiac preload that occurred during exsufflation. Then, EEXPO stops this cyclic impediment of venous return and allows cardiac preload to increase. Right cardiac preload increases first, and provided that the EEXPO is long enough for allowing the transit of this increase through the pulmonary vasculature, it is followed by the increase of left cardiac preload. The interruption of ventilation also stops the cyclic compression of the pulmonary vasculature, which facilitates the transference of preload increase from the right to the left side. The transient increase in cardiac preload induced by the EEXPO test can be seen as a small “self-preload challenge” which might be used to assess preload responsiveness [9].

A number of studies have now tested the reliability of the EEXPO test. Many were positive but some of them showed some contradicting results, which led us to perform a meta-analysis. Despite these studies, we report that the AUSROC of the EEXPO test to detect preload responsiveness is high, comparable to the one reported in meta-analyses for pulse pressure variation [32] and the passive leg raising test [5], and higher than the one found for the respiratory variations in the inferior or superior vena cava [33]. The present meta-analysis confirms another one recently published by Messina et al. [10], which included five less studies [18–22].

Importantly, the novelty of our meta-analysis is that it allowed us to investigate some of the factors which may, in theory, alter the test reliability and which have not been investigated in the former meta-analysis. First, no significant difference was observed between studies in which the duration of the respiratory hold was ≤ 15 s and studies in which it was longer, which indicates that a duration of 15 s appears enough. In practice, this is an important point since all ventilators do not allow respiratory holds ≥ 15 s.

Second, the level of PEEP might be theoretically important, since it is the level to which the airway pressure is reduced during EEXPO. However, in a previous study in which two levels of PEEP were compared in the same patients, the diagnostic accuracy of the EEXPO test was unchanged [19]. The present meta-analysis tends to confirm this, since the AUSROC was similar amongst studies with high or low PEEP levels. Nevertheless, both levels were defined according to the median value of PEEP levels, which was only 7 cmH_2_O. One should keep in mind that in theory, the haemodynamic effects of the EEXPO test should depend more on the respiratory driving pressure than on the PEEP alone, a hypothesis that remains to be tested. Of note, the worst reliability of the EEXPO test was reported by a study performed in prone positioning [15], in which the PEEP level was 8 cmH_2_O on average. Since there is no clear reason why prone positioning should change the reliability of the EEXPO test, and since this was reported in that single study, no clear conclusion about this point could be drawn without further investigations.

A third factor that might theoretically affect the EEXPO test reliability is the tidal volume. Two studies which have compared these two levels of tidal volume reported that diagnostic accuracy was correct at 8 mL/kg but poorer at 6 mL/kg [14, 21]. However, even if they did not directly compare different tidal volume levels, some of the other studies which reported excellent diagnostic accuracy had included some patients with low tidal volume values, as indicated by the mean and standard deviation reported in their whole population. If the test reliability had been poor in these patients, the averaged reliability could not have been so good. In line with these studies, the present meta-analysis did not show any difference in AUSROC when studies were compared with respect to the median of reported tidal volumes. These conflicting results suggest that the question to know whether the tidal volume actually influences the EEXPO test reliability is still unanswered.

A fourth and important issue is the method which is used for measuring the EEXPO-induced changes in CO. One advantage of the present meta-analysis was that it included studies using the devices that are the most used in the ICU nowadays [34]. As a matter of fact, the small threshold defining the test positivity may require precise CO monitoring devices. The least significant change of echocardiography [35] and oesophageal Doppler [20] is close to the diagnostic threshold of the EEXPO test. This is the reason why two studies performed with oesophageal Doppler [20] and echocardiography [16] have resolved this issue by combining the changes in CO induced by both end-expiratory and end-inspiratory holds. The present meta-analysis could not test the advantage of this strategy which was evaluated in these two studies only. However, even though the precision of pulse contour analysis [36] is higher than for the other tested methods, no significant difference has emerged when it was used to track CO changes compared to other methods. One study assessed the EEXPO effects through the changes in end-tidal carbon dioxide [12]. Of note, this way of tracking the EEXPO-induced changes in CO has been questioned [37]. However, the fact that the diagnostic accuracy of the EEXPO test is not influenced by the CO monitoring methods used is a strong argument in favour of the reliability of the test at bedside. Finally, the reliability of the EEXPO test was excellent in both the ICU and OR settings, but there is no obvious reason why it should not be the case.

The heterogeneity of the included studies is one of the limitations of our meta-analysis. However, the meta-regression analysis has investigated several possible sources of heterogeneity, identifying one of them (Additional file 1: Figure S5). Another limitation is that the studies included were all single-centre and enrolled a relatively small number of patients. Nevertheless, this is the interest of a meta-analysis to merge these small-size studies in order to draw more solid conclusions. Some of the studies suffered from biases as assessed with the QUADAS-2 (Additional file 1: Figure S3). Nevertheless, to improve investigation of their role as possible causes of heterogeneity, we performed a pre-specified subgroup analysis by dividing the studies according to the global risk of bias: no difference was observed in the accuracy of the EEXPO test between studies with overall lower and higher risk of bias. We also evaluated the overall quality of evidence of the studies included in the meta-analysis according to the GRADE system, with a whole judgement of “very low” (Additional file 1: Figure S3). Nonetheless, we believe that these findings should be extensible to each sample of EEXPO test studies, considering their recurrent weakness, related to small sample sizes, no power analysis and clinical heterogeneity. Finally, a large number of the included studies were performed by the same team, which had described the EEXPO test for the first time [8].

## Conclusion

This meta-analysis demonstrates that the EEXPO test is accurate in predicting fluid responsiveness both in the ICU and in the OR, regardless of the ventilatory settings and the duration of the expiratory hold. The accuracy is not different when the EEXPO test-induced changes on CO are detected by the pulse contour analysis or by other CO monitoring techniques.

## Supplementary information

**Additional file 1: Figure S1.** Searching strategy. **Figure S2.** Table showing continuity correction for diagnostic accuracy of the end-expiratory occlusion test in the including studies. **Figure S3.** Results of QUADAS-2 analysis. **Figure S4.** Overall quality assessment of the diagnostic accuracy of studies enrolled following the GRADE system **Figure S5.** Meta-regression analysis. **Figure S6.** Publication bias analysis. **Figure S7.** PRISMA checklist.

## Data Availability

The datasets used and/or analysed in the present study are available from the corresponding author on reasonable request.

## Abbreviations

AUROC: Area under the receiver operating characteristic
AUSROC: Area under the summary receiver operating characteristic
CO: Cardiac output
EEXPO: End-expiratory occlusion
ICU: Intensive care unit
OR: Operating room
PEEP: Positive end-expiratory pressure
PLR: Passive leg raising
