# Supplementary material for: The end-expiratory occlusion test for detecting preload responsiveness: a systematic review and meta-analysis
Source: Ann Intensive Care. 2020 May 24;10:65. doi: 10.1186/s13613-020-00682-8 (PMC7246264; doi:10.1186/s13613-020-00682-8)
Supplement: Supplementary file 1 — Additional file 1: Figure S1. Searching strategy. Figure S2. Table showing continuity correction for diagnostic accuracy of the end-expiratory occlusion test in the including studies. Figure S3. Results of QUADAS-2 analysis. Figure S4. Overall quality assessment of the diagnostic accuracy of studies enrolled following the GRADE system Figure S5. Meta-regression analysis. Figure S6. Publication bias analysis. Figure S7. PRISMA checklist. [file 13613_2020_682_MOESM1_ESM.docx]

# The end-expiratory occlusion test for detecting preload responsiveness: a systematic review and meta-analysis

Francesco GAVELLI, MD^1, 2, 3^; Rui SHI, MD^1, 2^; Jean-Louis TEBOUL, MD, PhD^1, 2^; Danila AZZOLINA, PhD^4^; Xavier MONNET, MD, PhD^1, 2^.

1 Service de médecine intensive-réanimation, Hôpital de Bicêtre, Hôpitaux Universitaires Paris-Saclay, Le Kremlin-Bicêtre, F-94270 France.

2 Université Paris-Saclay, Faculté de médecine Paris-Saclay, Inserm UMR S_999, Le Kremlin-Bicêtre, F-94270 France.

3 Emergency Medicine Unit, Department of Translational Medicine, Università degli Studi del Piemonte Orientale, 28100, Novara, Italy.

4 Department of Translational Medicine, Università degli Studi del Piemonte Orientale, 28100, Novara, Italy.

# Additional file 1

**Additional Figure S1 - Searching strategy**

#1 End expiratory occlusion

#2 End expiratory

#3 #1 OR #2

#4 fluid challenge

#5 volume expansion

#6 fluid administration

#7 volume challenge

#8 fluid responsiveness

#9 preload responsiveness

#10 #4 OR #5 OR #6 OR #7 OR #8 OR #9

#11 #4 AND #10

Example, PubMed:

((End[All Fields] AND ("exhalation"[MeSH Terms] OR "exhalation"[All Fields] OR "expiratory"[All Fields]) AND ("dental occlusion"[MeSH Terms] OR ("dental"[All Fields] AND "occlusion"[All Fields]) OR "dental occlusion"[All Fields] OR "occlusion"[All Fields])) OR (End[All Fields] AND ("exhalation"[MeSH Terms] OR "exhalation"[All Fields] OR "expiratory"[All Fields]))) AND ((((((preload[All Fields] AND responsiveness[All Fields]) OR (fluid[All Fields] AND responsiveness[All Fields])) OR (volume[All Fields] AND ("Challenge (Atlanta Ga)"[Journal] OR "challenge"[All Fields]))) OR (fluid[All Fields] AND ("organization and administration"[MeSH Terms] OR ("organization"[All Fields] AND "administration"[All Fields]) OR "organization and administration"[All Fields] OR "administration"[All Fields]))) OR (volume[All Fields] AND expansion[All Fields])) OR (fluid[All Fields] AND ("Challenge (Atlanta Ga)"[Journal] OR "challenge"[All Fields])))

**Additional Figure S2 – Continuity correction for diagnostic accuracy of the end-expiratory occlusion test in the including studies.**

|  | **Sensitivity** | **Sensitivity**  **95% CI** | **Specificity** | **Specificity**  **95% CI** | **False positive** | **True negative** | **False negative** | **True Positives** |
| --- | --- | --- | --- | --- | --- | --- | --- | --- |
| **Monnet et al.^8^** | 0.9 | 0.72 - 0.97 | 0.96 | 0.7 - 1 | 0 | 11 | 2 | 20.93 |
| **Monnet et al.^18^** | 0.97 | 0.78 - 1 | 0.89 | 0.7 - 0.97 | 2 | 20 | 0 | 17 |
| **Monnet et al.^11^** | 0.92 | 0.77 - 0.97 | 0.9 | 0.72 - 0.97 | 2 | 22 | 2 | 27.9 |
| **Silva et al.^19^** | 0.96 | 0.73 - 1 | 0.89 | 0.69 - 0.96 | 2 | 19 | 0 | 13 |
| **Guinot et al.^12^** | 0.81 | 0.64 - 0.91 | 0.7 | 0.45 - 0.87 | 4 | 10 | 5 | 22.96 |
| **Biais et al.^13^** | 0.98 | 0.81 - 1 | 0.8 | 0.59 - 0.91 | 4 | 17 | 0 | 20 |
| **Myatra et al.^14^** | 0.85 | 0.62 - 0.95 | 0.9 | 0.66 - 0.98 | 1 | 13 | 2 | 14.08 |
| **Yonis et al.^15^** | 0.34 | 0.16 - 0.59 | 0.97 | 0.79 - 1 | 0 | 18 | 10 | 4.95 |
| **Jozwiak et al.^16^** | 0.91 | 0.68 - 0.98 | 0.97 | 0.76 - 1 | 0 | 15 | 1 | 13.95 |
| **Georges et al.^17^** | 0.88 | 0.72 - 0.95 | 0.93 | 0.76 - 0.98 | 1 | 21 | 3 | 24.92 |
| **Dépret et al.^20^** | 0.83 | 0.58 - 0.95 | 0.9 | 0.66 - 0.98 | 1 | 13 | 2 | 12.04 |
| **Messina et al.^21^** | 0.89 | 0.69 - 0.96 | 0.82 | 0.61 - 0.93 | 3 | 16 | 2 | 18.774 |
| **Xu et al.^22^** | 0.8 | 0.64 - 0.9 | 0.91 | 0.79 - 0.97 | 3 | 36 | 7 | 29.16 |

Sensitivities and specificities have been reported. A continuity correction of 0.5 has been considered for computation. 95% Wilson confidence interval (CI) are also reported.

**Additional Figure S3. Results of QUADAS-2 analysis.**

|  | Patient selection | | Index test | | Reference standard | | Flow and timing |
| --- | --- | --- | --- | --- | --- | --- | --- |
|  | **Risk of bias** | **Applicability** | **Risk of bias** | **Applicability** | **Risk of bias** | **Applicability** | **Risk of bias** |
| Monnet et al.^8^ | ● | ● | ● | ● | ● | ● | ● |
| Monnet et al.^18^ | ● | ● | ● | ● | ● | ● | ● |
| Monnet et al.^11^ | ● | ● | ● | ● | ● | ● | ● |
| Silva et al.^19^ | ● | ● | ● | ● | ● | ● | ● |
| Guinot et al.^12^ | ● | ● | ● | ● | ● | ● | ● |
| Biais et al.^13^ | ● | ● | ● | ● | ● | ● | ● |
| Myatra et al.^14^ | ● | ● | ● | ● | ● | ● | ● |
| Yonis et al.^15^ | ● | ● | ● | ● | ● | ● | ● |
| Jozwiak et al.^16^ | ● | ● | ● | ● | ● | ● | ● |
| Georges et al.^17^ | ● | ● | ● | ● | ● | ● | ● |
| Dépret et al.^20^ | ● | ● | ● | ● | ● | ● | ● |
| Messina et al.^21^ | ● | ● | ● | ● | ● | ● | ● |
| Xu et al.^22^ | ● | ● | ● | ● | ● | ● | ● |

● : high; ● : low; ● : unclear.

Patient Selection

Risk of Bias (RB): Could the selection of patients have introduced bias? [Signaling question (SQ)1: Was a consecutive or random sample of patients enrolled? SQ2: Was a case-control design avoided? SQ3: Did the study avoid inappropriate exclusions?].

Index Test

RB: Could the conduct or interpretation of the index test have introduced bias? (SQ1: Were the index test results interpreted without knowledge of the results of the reference standard? SQ2: If a threshold was used, was it prespecified?).

Reference Standard

RB: Could the reference standard, its conduct, or its interpretation have introduced bias? (SQ1: Is the reference standard likely to correctly classify the target condition? SQ2: Were the reference standard results interpreted without knowledge of the results of the index test?).

Flow and Timing

RB: Could the patient flow have introduced bias? (SQ1: Was there an appropriate interval between the index test and reference standard? SQ2: Did all patients receive the same reference standard? SQ3: Were all patients included in the analysis?).

**Additional Figure S4 - Overall quality assessment of the diagnostic accuracy of studies enrolled following the GRADE system**

| **Quality assessment** | | | | | | | **Summary of findings** | | | | |
| --- | --- | --- | --- | --- | --- | --- | --- | --- | --- | --- | --- |
|  |  |  |  |  |  |  | **No. of patients** | | **Effect** | | **Quality** |
| **No. of studies** | **Design** | **Limitations** | **Indirectness of patients, intervention and comparator** | **Inconsistency** | **Imprecision** | **Other considerations** | **Preload responders^1^** | **Non preload responders^2^** | **Relative**  **(95% CI)** | **Absolute**  **(95% CI)^3^** |  |
| 13 studies (530 adult patients) | 13 prospective observational studies | Some limitations exist^4^ | Serious^5^ | Serious^6^ | Serious^7^ | The QUADAS-2 outcome suggested a high risk of bias for 2 studies^8^ | 276 | 254 | - | From 35.42 to 123.97 | ⊕○○○  VERY LOW |

^1^ Patients preload responders [True Positives (patients preload responders) and False Negatives (patients incorrectly classified has not being preload responders)].

^2^ Patients non preload responders [True Negatives (patients non preload responders) and False Positives (patients incorrectly classified has being non preload responders)].

^3^ 95% Confidence interval (CI) of the conventional pooled diagnostic odds ratio (DOR) calculated with MetaDisc

^4^ Short duration of the EEXPO test (1 study), low accuracy of the method used for hemodynamic monitoring (1 study).

^5^ Different cut-off values for preload responsiveness definition (≥10% in 3 studies, ≥15% in 10 studies).

^6^ Wide variation in the DOR estimates 95% Cis, I^2^ for sensitivity 70.8% with p<0.001.

^7^ Small samples size; one-centre studies.

^8^ For more details, see Additional material S2.

Each domain was evaluated according to Ryan R, Hill S (2016) How to GRADE the quality of the evidence. Cochrane Consumers and Communication Group. Version 3.0 December 2016. Available on: [http://cccrg.cochrane.org/author-resources](http://cccrg.cochrane.org/author-resources.%20Version%203.0%20December%202016) (last access: June 15, 2018). The table structure and quality of evidence were showed according to Schünemann H, Brożek J, Guyatt G, Oxman A (2013) GRADE handbook for grading quality of evidence and strength of recommendations. The GRADE Working Group. Available on: https://gdt.gradepro.org/app/handbook/handbook.html (last access: May 19, 2018), and Guyatt GH, Oxman AD, Santesso N, Helfand M, Vist G, Kunz R, Brozek J, Norris S, Meerpohl J, Djulbegovic B, Alonso-Coello P, Post PN, Busse JW, Glasziou P, Christensen R, Schünemann HJ (2013) GRADE guidelines: 12. Preparing Summary of Findings tables - binary outcomes. J Clin Epidemiol 66:158-172 (doi: 10.1016/j.jclinepi.2012.01.012).

**Additional Figure S5– Metaregression analysis**

**Additional Figure S5.1 – Metaregression analysis on Tidal Volume (≤7 *vs.* >7 mL/kg)**

|   Panel A – Bivariate Model AUC estimate and SROC curve for the Reitsma et al. (2005) model. Separate model Estimated AUC for Tidal volume ≤7 mL/kg is 0.96 [0.92 - 0.97] and for Tidal volume >7 mL/kg is 0.89 [0.82-0.95].  Bootstrap p-value for difference in separate AUC estimates is 0.44. |
| --- |
| \|  \| **Log OR** \| **p value** \| **95% LB** \| **95% UB** \| \| --- \| --- \| --- \| --- \| --- \| \| **Sens** \| -0.015 \| 0.982 \| -1.321 \| 1.29 \| \| **FPR** \| 0.463 \| 0.339 \| -0.486 \| 1.412 \|   Panel B – Metaregression. Tidal volume adjusted bivariate model. Log OR covariate effects on sensitivity and FPR (1-specificity) have been reported with 95% CI.  The p-value for the Likelihood ratio test considering the Metaregression model in comparison with a model without covariate is 0.68. |

**Additional Figure S5.2 – Metaregression analysis on haemodynamic monitoring (pulse contour analysis vs other methods)**

| **  *Panel A – Bivariate Model AUC estimate and SROC curve for the Reitsma et al. (2005) model. Separate model Estimated AUC for pulse contour analysis is 0.93 [0.91-0.95] and for other monitoring methods is 0.87 [0.82-0.96].*  *Bootstrap p-value for difference in separate AUC estimates is 0.62.* |
| --- |
| \|  \| **Log OR** \| **p value** \| **95% LB** \| **95% UB** \| \| --- \| --- \| --- \| --- \| --- \| \| **Sens** \| 0.08 \| 0.852 \| -0.851 \| 1.03 \| \| **FPR** \| -0.401 \| 0.41 \| -1.355 \| 0.552 \|   *Panel B – Metaregression. Hemodynamic monitoring adjusted bivariate model. Log OR covariate effects on sensitivity and FPR (1-specificity) have been reported with 95% CI.*  *The p-value for the Likelihood ratio test considering the Metaregression model in comparison with model without covariate is 0.683.* |

**Additional Figure S5.3 – Metaregression analysis on EEXPO duration (≤15 seconds *vs.* >15 seconds)**

| **  *Panel A – Bivariate Model AUC estimate and SROC curve for the Reitsma et al. (2005) model. Separate model Estimated AUC for EEXPO ≤15 seconds is 0.93 [0.90-0.96] and for EEXPO > 15 seconds is 0.93 [0.88-0.95].*  *Bootstrap p-value for difference in separate AUC estimates is 0.2.* |
| --- |
| \|  \| **Log OR** \| **p value** \| **95% LB** \| **95% UB** \| \| --- \| --- \| --- \| --- \| --- \| \| **Sens** \| 0.203 \| 0.792 \| -1.305 \| 1.712 \| \| **FPR** \| 0.328 \| 0.493 \| -0.611 \| 1.268 \|   *Panel B – Metaregression. EEXPO duration adjusted bivariate model. Log OR covariate effects on sensitivity and FPR (1-specificity) have been reported with 95% CI.*  *The p-value for the Likelihood ratio test considering the Metaregression model in comparison with model without covariate is 0.786.* |

**Additional Figure S5.4 – Metaregression analysis on PEEP level (≤7 *vs.* >7 cmH_2_O)**

| **  *Panel A – Bivariate Model AUC estimate and SROC curve for the Reitsma et al. (2005) model. Separate model Estimated AUC for PEEP values ≤7 cmH_2_O is 0.89 [0.83-0.95] and* *0.95 [0.92-0.97] for PEEP values >7 cmH_2_O.*  *Bootstrap p-value for difference in separate AUC estimates is 0.386.* |
| --- |
| \|  \| **Log OR** \| **p value** \| **95% LB** \| **95% UB** \| \| --- \| --- \| --- \| --- \| --- \| \| **Sens** \| -0.418 \| 0.52 \| -1.691 \| 0.855 \| \| **FPR** \| -0.783 \| 0.11 \| -1.743 \| 0.177 \|   *Panel B – Metaregression. PEEP level adjusted bivariate model. Log OR covariate effects on sensitivity and FPR (1-specificity) have been reported with 95% CI.*  *The p-value for the Likelihood ratio test considering the Metaregression model in comparison with model without covariate is 0.683.* |

**Additional Figure S5.5 – Metaregression analysis on EEXPO setting (OR *vs*. ICU)**

|   Panel A – Bivariate Model AUC estimate and SROC curve for the Reitsma et al. (2005) model. Separate model Estimated AUC for ICU setting is 0.95 [0.93-0.96] and 0.86 [0.82-0.93] for OR setting.  Bootstrap p-value for difference in separate AUC estimates is 0.66. |
| --- |
| \|  \| **Log OR** \| **P value** \| **95% LB** \| **95% UB** \| \| --- \| --- \| --- \| --- \| --- \| \| **Sens** \| -0.085 \| 0.903 \| -1.446 \| 1.276 \| \| **FPR** \| 0.908 \| 0.036 \| 0.058 \| 1.757 \|   Panel B – Metaregression. Setting adjusted bivariate model. Log OR covariate effects on sensitivity and FPR (1-specificity) have been reported with 95% CI.  The p-value for the Likelihood ratio test, considering the Metaregression model in comparison with a model without covariate, is 0.09. |

**Additional Figure S5.6 – Metaregression analysis on Risk of Bias (Overall lower *vs.* Overall higher)**

| **  *Panel A – Bivariate Model AUC estimate and SROC curve for the Reitsma et al. (2005) model. Separate model Estimated AUC for overall lower risk of bias is 0.96 [0.92-0.97] and is* *0.91 [0.83-0.95] for overall higher risk of bias.*  *Bootstrap P-Value for difference in separate AUC estimates is 0.45.* |
| --- |
| \|  \| **Log OR** \| **p value** \| **95% LB** \| **95% UB** \| \| --- \| --- \| --- \| --- \| --- \| \| **Sens** \| -1.167 \| 0.051 \| -2.339 \| 0.005 \| \| **FPR** \| 0.514 \| 0.281 \| -0.419 \| 1.446 \|   *Panel B – Metaregression. Risk of bias adjusted bivariate model. Log OR covariate effects on sensitivity and FPR (1-specificity) have been reported with 95% CI.*  *The p-value for the Likelihood ratio test considering the Metaregression model in comparison with model without covariate is 0.049.* |

**Additional Figure S6 – Publication bias**

|  | **Coefficient** | **Standard error** | **t** | **p > t** | **95% Lower CI** | **95% Upper CI** |
| --- | --- | --- | --- | --- | --- | --- |
| **Biais** | 2.45027 | 13.93579 | 0.18 | 0.864 | -28.22195 | 33.123 |
| **Intercept** | 3.774129 | 2.137803 | 1.77 | 0.105 | -.0931142 | 8.479401 |

CI : confidence interval.

**Additional Figure S7 – PRISMA Checklist**

| **Section/topic** | **#** | **Checklist item** | **Reported on page #** |
| --- | --- | --- | --- |
| **TITLE** | | |  |
| Title | 1 | Identify the report as a systematic review, meta-analysis, or both. | 1 |
| **ABSTRACT** | | |  |
| Structured summary | 2 | Provide a structured summary including, as applicable: background; objectives; data sources; study eligibility criteria, participants, and interventions; study appraisal and synthesis methods; results; limitations; conclusions and implications of key findings; systematic review registration number. | 2 |
| **INTRODUCTION** | | |  |
| Rationale | 3 | Describe the rationale for the review in the context of what is already known. | 3-4 |
| Objectives | 4 | Provide an explicit statement of questions being addressed with reference to participants, interventions, comparisons, outcomes, and study design (PICOS). | 4 |
| **METHODS** | | |  |
| Protocol and registration | 5 | Indicate if a review protocol exists, if and where it can be accessed (e.g., Web address), and, if available, provide registration information including registration number. | 5 |
| Eligibility criteria | 6 | Specify study characteristics (e.g., PICOS, length of follow-up) and report characteristics (e.g., years considered, language, publication status) used as criteria for eligibility, giving rationale. | 4-5 |
| Information sources | 7 | Describe all information sources (e.g., databases with dates of coverage, contact with study authors to identify additional studies) in the search and date last searched. | 5 |
| Search | 8 | Present full electronic search strategy for at least one database, including any limits used, such that it could be repeated. | Supplemental S1 |
| Study selection | 9 | State the process for selecting studies (i.e., screening, eligibility, included in systematic review, and, if applicable, included in the meta-analysis). | 5-6 |
| Data collection process | 10 | Describe method of data extraction from reports (e.g., piloted forms, independently, in duplicate) and any processes for obtaining and confirming data from investigators. | 5-6 |
| Data items | 11 | List and define all variables for which data were sought (e.g., PICOS, funding sources) and any assumptions and simplifications made. | 5 |
| Risk of bias in individual studies | 12 | Describe methods used for assessing risk of bias of individual studies (including specification of whether this was done at the study or outcome level), and how this information is to be used in any data synthesis. | 6 |
| Summary measures | 13 | State the principal summary measures (e.g., risk ratio, difference in means). | 6-7 |
| Synthesis of results | 14 | Describe the methods of handling data and combining results of studies, if done, including measures of consistency (e.g., I^2^) for each meta-analysis. | 7 |
| Risk of bias across studies | 15 | Specify any assessment of risk of bias that may affect the cumulative evidence (e.g., publication bias, selective reporting within studies). | 7-8 |
| Additional analyses | 16 | Describe methods of additional analyses (e.g., sensitivity or subgroup analyses, meta-regression), if done, indicating which were pre-specified. | 7-8 |
| **RESULTS** | | |  |
| Study selection | 17 | Give numbers of studies screened, assessed for eligibility, and included in the review, with reasons for exclusions at each stage, ideally with a flow diagram. | 8 |
| Study characteristics | 18 | For each study, present characteristics for which data were extracted (e.g., study size, PICOS, follow-up period) and provide the citations. | 8  Table 1, 2 |
| Risk of bias within studies | 19 | Present data on risk of bias of each study and, if available, any outcome level assessment (see item 12). | 11  Supplemental S3, S4 |
| Results of individual studies | 20 | For all outcomes considered (benefits or harms), present, for each study: (a) simple summary data for each intervention group (b) effect estimates and confidence intervals, ideally with a forest plot. | Table 3 |
| Synthesis of results | 21 | Present results of each meta-analysis done, including confidence intervals and measures of consistency. | 9  Supplemental S2 |
| Risk of bias across studies | 22 | Present results of any assessment of risk of bias across studies (see Item 15). | 11  Supplemental S6 |
| Additional analysis | 23 | Give results of additional analyses, if done (e.g., sensitivity or subgroup analyses, meta-regression [see Item 16]). | 9-11  **Additional Figure** S5 |
| **DISCUSSION** | | |  |
| Summary of evidence | 24 | Summarize the main findings including the strength of evidence for each main outcome; consider their relevance to key groups (e.g., healthcare providers, users, and policy makers). | 12-14 |
| Limitations | 25 | Discuss limitations at study and outcome level (e.g., risk of bias), and at review-level (e.g., incomplete retrieval of identified research, reporting bias). | 14-15 |
| Conclusions | 26 | Provide a general interpretation of the results in the context of other evidence, and implications for future research. | 15 |
| **FUNDING** | | |  |
| Funding | 27 | Describe sources of funding for the systematic review and other support (e.g., supply of data); role of funders for the systematic review. | 17 |

*From:*  Moher D, Liberati A, Tetzlaff J, Altman DG, The PRISMA Group (2009). Preferred Reporting Items for Systematic Reviews and Meta-Analyses: The PRISMA Statement. PLoS Med 6(6): e1000097. doi:10.1371/journal.pmed1000097
